# Cepharanthine Attenuates Early Brain Injury after Subarachnoid Hemorrhage in Mice via Inhibiting 15-Lipoxygenase-1-Mediated Microglia and Endothelial Cell Ferroptosis

**DOI:** 10.1155/2022/4295208

**Published:** 2022-02-09

**Authors:** Shiqi Gao, Liuzhi Zhou, Jianan Lu, Yuanjian Fang, Haijian Wu, Weilin Xu, Yuanbo Pan, Junjie Wang, Xiaoyu Wang, Jianmin Zhang, Anwen Shao

**Affiliations:** ^1^Department of Neurosurgery, The Second Affiliated Hospital, Zhejiang University School of Medicine, Hangzhou, China; ^2^Zhejiang University School of Medicine, Hangzhou, China; ^3^Brain Research Institute, Zhejiang University, Hangzhou, Zhejiang, China; ^4^Collaborative Innovation Center for Brain Science, Zhejiang University, Hangzhou, Zhejiang, China

## Abstract

**Background:**

Ferroptosis is a newly identified form of programmed cell death caused by iron-dependent lipid peroxidation. Our study was designed to determine the expression patterns and role of 15-lipoxygenase-1 (ALOX15) in subarachnoid hemorrhage (SAH) and to investigate whether cepharanthine (CEP) can inhibit ferroptosis by inhibiting ALOX15 in specific cell types.

**Methods:**

A mouse model of SAH was developed by the endovascular perforation method. bEend.3 endothelial cells and BV2 microglial cells as well as RSL3 and hemin were used to simulate SAH *in vitro*. Mice and cell lines were treated with CEP and a group of specific oxygenase inhibitors to explore the protection effect from ferroptosis. Lipid peroxidation staining with BODIPY 581/591 C11 and transmission electron microscopy were used to identify ferroptosis *in vitro* and *in vivo*.

**Results:**

In the present study, the accumulation of lipid peroxide, a defect in the glutathione peroxidase 4 (GPx4)/glutathione (GSH) antioxidant system, highly expressed ALOX15 in microglia and endothelium, and ferroptotic changes in microglial mitochondria confirmed the occurrence of ferroptosis after SAH *in vivo*. Further, CEP was shown to inhibit ferroptosis and improve neurological function by downregulating the expression of ALOX15. During *in vitro* experiments, we investigated the important role ALOX15 in RSL3-induced endothelial ferroptosis. In addition, we found that M2-type microglia are more sensitive to RSL3-induced ferroptosis than M1-type microglia and that hemin probably induced ferroptosis in M2-type microglia by increasing ALOX15 levels and decreasing GPx4 levels. The effect of CEP treatment was also demonstrated *in vitro*.

**Conclusions:**

In summary, to the best of our knowledge, this is the first study demonstrating that ferroptosis occurred in the microglia and endothelium after SAH, and this process was facilitated by increased ALOX15 levels. More importantly, treatment with CEP could inhibit ferroptosis through downregulating the expression of ALOX15.

## 1. Introduction

Subarachnoid hemorrhage (SAH) is a life-threatening disease mainly caused by intracranial aneurysm rupture, which accounts for 5%–10% of all stroke subtypes. Despite great efforts being made, it still accounts for high mortality and morbidity [1]. Early brain injury (EBI), which occurs within the first 72 h following SAH, has been widely accepted as the key factor responsible for a poor prognosis of SAH patients [2, 3]. Many pathophysiological events are involved in EBI, such as blood–brain barrier (BBB) disruption, neuroinflammation, lipid peroxidation, and various types of cell death [4, 5]. Treatments aiming at these events are promising interventions in SAH research.

Cell death, especially apoptosis, has been demonstrated as a hallmark after SAH during the past few decades [6, 7]. Ferroptosis, characterized by iron-dependent lipid peroxidation, is a new form of programmed cell death, which differs from apoptosis or necroptosis [8, 9]. The morphological features of a ferroptotic cell are shrunken mitochondria with increased mitochondrial membrane density, a reduction of mitochondria crista, and outer membrane rupture [8, 10]. Ferroptosis has been recently reported in the pathological process of hemorrhagic stroke including intracerebral hemorrhage (ICH) and SAH [11–15]. However, for specific ferroptotic cell types, previous studies in hemorrhagic stroke mainly focus on neurons, and the detailed mechanisms are still unclear. Given that specific inhibitors of ferroptosis ferrostatin-1 (Fer-1) and liproxstatin-1 (lip-1) can protectively reduce brain edema and neuroinflammation after SAH [13, 14], it seems meaningful to explore ferroptosis in other types of cells such as endothelial cells and microglia.

Furthermore, 15-lipoxygenase-1 (ALOX15), also known as 12/15-lipoxygenase (12/15-LOX), is a non-heme, iron-containing fatty acid dioxygenases that is constitutively expressed in reticulocytes, alveolar macrophages, airway epithelial cells, vascular cells, and so on [16]. ALOX15 can metabolize several polyunsaturated fatty acids (PUFAs) to form biologically active lipid mediators. Moreover, 12(S)-hydroxyicosatetraenoic (12(S)-HETE) acid and 15(S)-hydroxyicosatetraenoic acid (15(S)-HETE), the principle metabolites of arachidonic acid (AA) catalyzed by ALOX15, have been implicated in various physiological and pathological processes [16, 17]. For example, they regulate vascular reactivity and anti- or proinflammation in a variety of mammalian cells in a concentration-dependent manner [17]. Recently, several studies have demonstrated that ferroptotic signals are generated by ALOX15 directly oxidizing AA- and adrenoyl- (AdA-) phosphatidylethanolamines (PE) [18, 19]. This death signal is initiated at the cell membrane and can be further amplified in the absence of glutathione peroxidase 4 (GPx4). ALOX15-mediated cell death and neuroinflammation have been observed in many central nervous system (CNS) diseases, such as ischemic stroke, ICH, multiple sclerosis (MS), and traumatic brain injury (TBI) [20–23]. In particular, the application of the specific ALOX15 inhibitor baicalein has been shown to suppress ferroptosis in posttraumatic epilepsy and TBI mouse models [20, 24]. However, studies on ALOX15 are pretty rare with regard to SAH.

Cepharanthine (CEP) is a type of bisbenzylisoquinoline (BBIQ) alkaloid, isolated from Chinese herbal medicine [25]. As a natural medicine mainly used to treat leukopenia, snake bites, xerostomia, and alopecia, CEP has proven its neuroprotective effects because of the anti-inflammatory and antioxidative properties and its ability to cross the BBB [25]. Moreover, it was reported to alleviate oxidation and inflammation after cerebral ischemia/reperfusion injury by reducing ALOX15 expression [26]. Based on the structure of biamine moieties in CEP molecules, it also shows antilipid peroxidation activity benefit from its direct radical scavenging activity [27]. Similar to EDTA, CEP can effectively chelate ferrous ions (Fe^2+^). The amphipathic nature of CEP facilitates its permeability through the cell membrane and interaction with lipophilic compounds [25]. More recently, another BBIQ, dauricine, was reported to inhibit ferroptosis after ICH by loading with a nanocarrier [28]. Accordingly, we assume that CEP is another potential drug to alleviate ferroptosis and provide neuroprotection via the inhibition of ALOX15 expression. To the best of our knowledge, the pharmacological action and molecular mechanism of CEP in SAH has never been investigated.

In the present study, we explored the role of CEP and ALOX15 in SAH. The results showed that CEP may attenuate the neurological deficits by inhibiting ferroptosis in endothelial cells and microglia with the help of ALOX15 after SAH. Furthermore, the sensitivity of microglia of different polarization types to ferroptosis and the potentially involved proteins were also investigated in this study.

## 2. Materials and Methods

### 2.1. Animals

**Figure 1:**
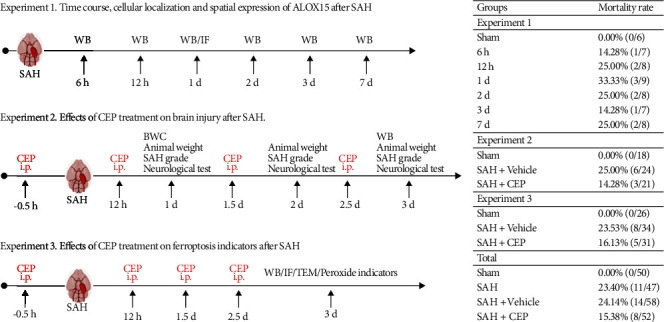
Experimental design, animal groups, and mortality.

All experimental procedures were warranted by the Institutional Ethics Committee of the Second Affiliated Hospital, Zhejiang University School of Medicine. The procedures were conducted according to National Institutes of Health guidelines for the Care and Use of laboratory Animals in Neuroscience Research and ARRIVE guidelines. Adult, male C57/BL6 mice weighing 20-25 g were used in this study. The mice were housed in a temperature- and humidity-controlled room under a standard 12 h light/dark cycle with free access to food and water. And no significant changes in those physiological variables were noted among the different groups (Figure 1).

### 2.2. SAH Model and Drug Administration

The endovascular perforation model, as previously described, was adopted [11]. General anesthesia was induced by peritoneal injection with pentobarbital (40 mg/kg) before the carotid artery, and its bifurcation were necessarily exposed. A sharp 5-0 monofilament nylon thread was inserted into the left internal carotid artery from the external carotid artery, and then, the thread perforated the vessel at the bifurcation of the anterior cerebral arteries and middle cerebral arteries. The thread was withdrawn after 4 repeated punctures. Apart from the blood vessel wall perforating, mice in the sham group underwent the same surgical procedures. Mice were randomly divided into four groups: (1) sham, (2) SAH, (3) SAH+vehicle, and (4) SAH+CEP. Vehicle (30% sodium carboxymethylcellulose, Aladdin Regents, Shanghai, China) and CEP (20 mg/kg, MCE, NJ, USA) were intraperitoneally injected 30 min before SAH induction and supplemented every 12 h before sacrifice (Figure 1). The degree of SAH was quantitatively assessed through a new grading system whose total score was rated as mild (0–7), moderate (8–12), and severe (13–18) [27].

### 2.3. Neurological Assessment and Animal Weight

Neurological function was assessed by the modified Garcia scoring system as described previously [28]. The total Garcia score was graded with a scale ranging from 1 to 18 which was composed of 6 individual trials: spontaneous activity (0-3), symmetry of limbs (0-3), forelimb extension (0-3), climbing (1-3), body proprioception (1-3), and reaction to vibrissae (1-3). Lower scores indicated worse neurological performance. The modified Garcia scoring and mouse weight were evaluated at 1 h before surgery and 1 day, 2 days, and 3 days after SAH induction.

### 2.4. Brain Water Content

We evaluated the brain water content at 24 h after SAH through the wet-dry method. Briefly, brains of mice were collected immediately after being sacrificed. Then, the brains were separated into four parts: the left hemisphere, right hemisphere, cerebellum, and brain stem, and each part was immediately weighed for the wet weight. After being left in an electrothermostatic oven for 72 h (105°C, dry weight), each part was subsequently weighed for the dry weight. Finally, we calculated the brain water content according to the formula for the wet-dry method: [(wet weight − dry weight)/(wet weight)] × 100%.

### 2.5. Measurement of Oxidative Stress Landmark

The contents of malondialdehyde (MDA), 4-hydroxynonenal (4-HNE), and glutathione (GSH) at 3 days after SAH were detected with commercial kits (MDA: #A003; 4-HNE: #H268; GSH: #A006, Jiancheng Bioengineering Institute, Nanjing, China). Briefly, the injured (left) temporal cortex was immediately homogenized at a low temperature with the reagents included in the kits. After determining the protein concentration of each homogenate with a BCA protein assay kit (Thermo Fisher Scientific, MA, USA), MDA, 4-HNE, and GSH were measured according to the manufacturer's instructions. The results were normalized with protein content to ensure the comparability within different groups.

### 2.6. Western Blot Analysis

The injured (left) temporal cortex was collected from sacrificed mice at 1 day or 3 days after SAH, or sham operation, and stored in liquid nitrogen for subsequent processes. Brain samples were homogenized in RIPA lysis buffer and further centrifuged at 14,000 g at 4°C for 20 min. After determining the protein concentration with a BCA protein assay kit (Thermo Fisher Scientific, MA, USA), we resuspended the protein solution with a loading buffer and then denatured the mixture at 100°C for 8 min to obtain standardized protein samples (40 *μ*g/*μ*L). Equal amounts of protein (40 *μ*g) were loaded onto 12% SDS-PAGE gel and then transferred to the PVDF membrane after separating by electrophoresis. Next, membranes were blocked with 5% nonfat blocking grade milk and incubated with the following primary antibodies overnight at 4°C: mouse anti-*β*-actin (1 : 10000, 66009, Proteintech), rabbit anti-ALOX15 (1 : 1000, ab244205, Abcam), rabbit anti-ZO-1 (1 : 1000, 21773, Proteintech), rabbit antioccludin (1 : 1000, 27260, Proteintech), rabbit anti-MMP-9 (1 : 500, 10375, Proteintech), rabbit anti-GPx4 (1 : 1000, a11243, ABclonal), rabbit anti-SLC7A11 (xCT) (1 : 1000, 26864, Proteintech), and rabbit anti-HO-1 (1 : 10000, ab68477, Abcam). The membranes were incubated with the respective horseradish peroxidase-conjugated secondary antibodies at 1 : 10000 dilutions for 1 h at room temperature. Finally, the protein was visualized with the ECL plus chemiluminescence reagent kit (Amersham Bioscience, Arlington Heights, IL). The results were normalized using *β*-actin as an internal control.

### 2.7. Immunofluorescence Staining

After deep anesthetization, the mouse underwent a transcardiac perfusion of 20 mL of chilled PBS (0.01 M, pH 7.4), followed by another 20 mL of perfusion with 4% paraformaldehyde (PFA). The whole brain was removed rapidly to be preserved in 4% PFA solution at 4°C for postfixation for 24-48 h and then replaced into a sucrose solution (30%) for complete dehydration for 72 h. Next, the brain specimens were coronally sliced into 8 *μ*m sections, which were to be mounted on a glass slide for immunofluorescence and Fluoro-Jade C staining.

For immunofluorescence staining, slides were reheated for 30 min at room temperature and rinsed with PBS three times and then blocked with QuickBlotTM Blocking Buffer for immunofluorescence (Beyotime, Shanghai, China) for 60 min at room temperature. Next, slides were incubated with the following primary antibodies (12 h, 4°C): anti-NeuN (1 : 500, ab104224, Abcam, Cambridge, UK), anti-Iba-1 (1: 200, ab5076, Abcam, Cambridge, UK) and anti-GFAP (1 : 500, ab10062, Abcam, Cambridge, UK), anti-CD31 (1 : 100, AF3628, R&D Systems, MN, USA), anti-ALOX15 (1 : 150, sc-133085, Santa Cruz, TX, USA), and anti-MPO (1 : 200, ab225474, Abcam, Cambridge, UK). After that, the slides were rinsed again for the incubation (2 h, 25°C) with corresponding fluorescence-conjugated secondary antibodies and covered with Fluoroshield Mounting Medium with DAPI (ab104139, Abcam, Cambridge, UK). Finally, images were taken using a fluorescence microscope (Olympus Co., Tokyo, Japan).

### 2.8. Transmission Electron Microscopy (TEM)

Mice were sacrificed and perfused as described in the immunofluorescence staining process. Fragments (~1mm^3^) were separated from the left temporal cortex and immersed in 2.5% glutaraldehyde at 4°C for 8 h. Samples were preprocessed as previously described [12]. After being refixed in 1% osmium tetroxide for 1 h, samples were stained in 2% uranyl acetate and then dehydrated in graded ethanol. Next, samples were embedded in 100% acetone for 4 h, cut into 100 nm ultrathin sections, and finally stained with 4% uranyl acetate and 0.5% lead citrate. The ultrastructure of samples was observed using a TEM (Philips Tecnai 10, Netherlands).

### 2.9. Cell Lines and Cell Treatment

The mouse microglial cell line BV2 and the mouse brain microvascular endothelial cells bEnd.3 were cultured at 37°C with 5% CO_2_ in Dulbecco's modified Eagle's medium with 10% fetal bovine serum, 100 U/mL penicillin, and 100 *μ*g/mL streptomycin. (1S,3R)-RSL3 (0.2 *μ*M–4 *μ*M, MCE, NJ, USA) was used to simulate the condition of GPX4 absence, which was also deemed as the *in vitro* model of ferroptosis in BV2 and bEnd.3. Cotreatment with CEP (0.5-5 *μ*g/mL, MCE), aspirin (277.5 *μ*M, 10 × IC50, MCE), celecoxib (400 nM, 10 × IC50, MCE), zileuton (5 *μ*M, MCE), PD146176 (1.08 *μ*M, 2 × IC50, Santa Cruz), ML351 (400 nM, 2 × IC50, Topscience), baicalein (6.24 mM, 2 × IC50, MCE), and ML355 (0.68 *μ*M, 2 × IC50, Topscience) were introduced to evaluate the protective effects of CEP and specific oxygenase inhibitor against RSL3-induced bEnd.3 ferroptosis. Lipopolysaccharide (LPS, Sigma-Aldrich, MO, USA) and IL-4/IL-13 (Sino Biological, Beijing, China) were, respectively, used to induce M1 or M2 type of BV2 cells [29]. Briefly, 24 h after planting, cells were cultured in complete medium containing LPS at 100 ng/mL final concentration for 24 h to induce the M1 phenotype of BV-2 cells and 10 ng/mL final concentration of IL-4 and IL-13 to induce the M2 phenotype. Then, we stimulated BV2 cells with 150 *μ*M hemin (Sigma-Aldrich, MO, USA) to simulate the SAH model *in vitro*.

### 2.10. Cell Viability

The viability of BV2 and bEnd.3 cells after treatments was determined with a cell counting kit-8 (CCK-8, Boster, Wuhan, China). Briefly, cell suspensions (30000/mL) were planted in a 96-well plate and cultured overnight. After exposure of the treatment for 24 h, every well of cells was applied with a mixture containing 90 *μ*L medium and 10 *μ*L CCK-8 reagent. Then, the optical density at 450 nm of the plate was measured after incubation at 37°C for 1 h.

### 2.11. Lipid Peroxide Fluorescence Staining

Lipid peroxidation in cells was determined with the live cell analysis reagent BODIPY 581/591 C11 (D3861, Thermo Fisher Scientific, MA, USA). Differently treated cells were washed with PBS for two times, then were incubated with a 10 *μ*M kit reagent for 30 min in an incubator. Upon lipid peroxidation, the fluorescence excitation wavelength of cells shifted from 581 (red) to 500 (green). Images were acquired with a fluorescence microscope under unified parameters. The fluorescence ratio of green/red was analyzed with ImageJ software (ImageJ 1.4, NIH, USA).

### 2.12. Statistical Analysis

Continuous data are presented as mean ± standard deviation (SD) or median (interquartile range) based on the normality and homogeneity of variance. For those normal distributed data, significant differences among groups were analyzed using Student's *t* test (2 groups) and one-way analysis of variance (ANOVA) (≥3 groups). For the data that do not conform to normal distribution, comparison among groups was conducted by Mann–Whitney *U* test (2 groups) or Kruskal-Wallis test (≥3 groups). Two-way repeated-measure ANOVA followed by Tukey's post hoc test was used to analyze the persistent neurological functions. Statistical analysis was performed using GraphPad Prism 8.1 and SPSS 23.0 software. A value of *p* < 0.05 was considered as significant.

## 3. Results

### 3.1. Time Course, Cellular Localization, and Spatial Expression of ALOX15

**Figure 2:**
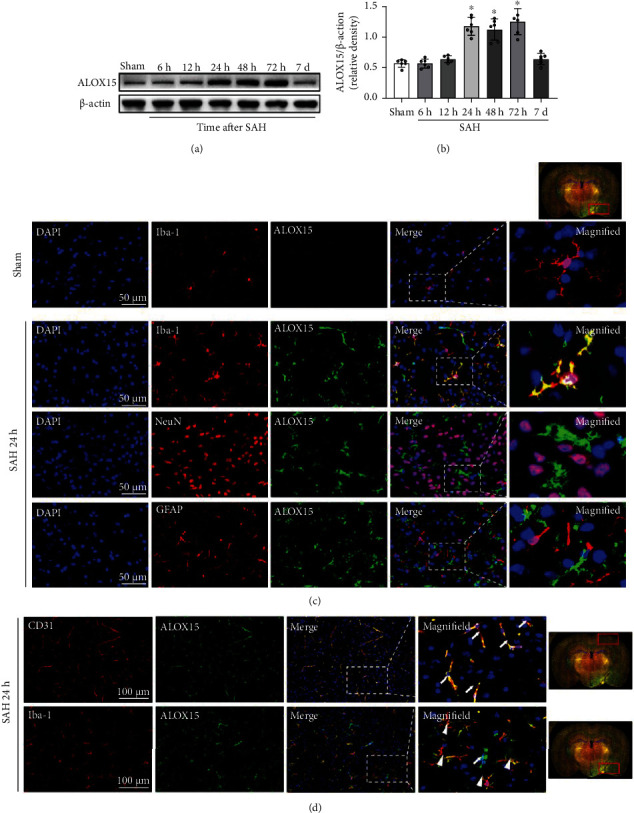
Times course, spatial expression, and cellular localization of ALOX15 after SAH. (a, b) Representative Western blot images and densitometric quantification of time-dependent expression of ALOX15 after SAH. Data are expressed as mean ± SD, ^∗^*p* < 0.05 vs. sham group, *n* = 6 per group. (c, d) Representative photographs of colocalization of ALOX15 (green) with neurons (NeuN, red), astrocytes (GFAP, red), microglia (Iba-1, red), and endothelial cell (CD31, red) in the left hemisphere (injury ipsilateral) at 24 h after SAH modeling. Nuclei were stained with DAPI (blue). White arrow represents ALOX15 colocalizing with microvascular endothelium, and white triangle represents ALOX15 colocalizing with microglia. Right whole brain scan image indicates the location of staining in the brain (small red box), scale bar = 50 *μ*m, *n* = 3 per group.

Western blotting was performed to evaluate the time course for ALOX15 protein levels. The results showed that the level of ALOX15 significantly rose at 24 h after SAH induction and remained at a high level until 3 days, after which the expression of ALOX15 dropped to normal levels at 7 days after SAH (Figures 2(a) and 2(b)). Based on this result, the time point of 1 day or 3 days after SAH incidence was identified as the main observation time points in subsequent experiments.

Double immunofluorescence staining of ALOX15 with neurons (NeuN), microglia (Iba-1), astrocytes (GFAP), and endothelial cells (CD31) was performed in the sham group and SAH groups 24 h after modeling. Results suggested that ALOX15 was mainly expressed in microglia and endothelial cells (Figures 2(c) and 2(d)), rather than in neurons or astrocytes. In contrast, almost no immunofluorescence was detectable in the sham group (Figure 2(c)). Furthermore, in the temporal cortex close to the blood clot, ALOX15 was mainly expressed in microglia, whereas it was mainly expressed in the endothelial cells in the distal cortex or basal ganglia away from the blood clot **(**Figure 2(d)).

### 3.2. Effects of CEP Treatment on SAH Grade, Neurological Outcomes, Brain Edema, and Animal Weight at 1 Day to 3 Days after SAH

**Figure 3:**
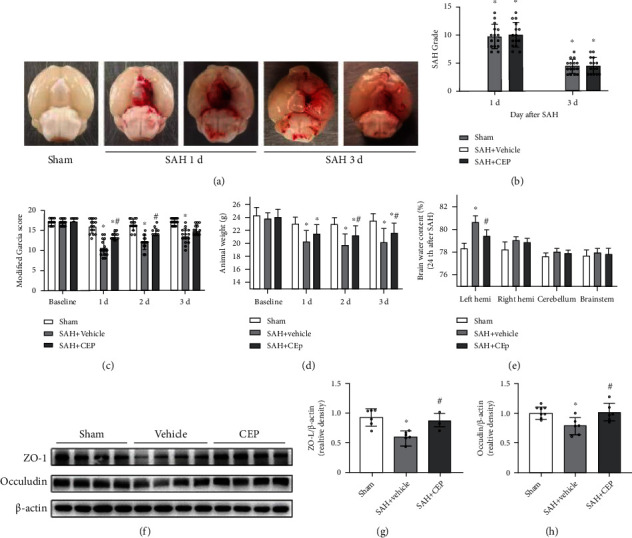
The effects CEP on SHA grade, neurological performance, brain edema, and BBB disruption after SAH. (a) Representative brain images from the SAH+vehicle and SAH+CEP groups. (b) The quantification of SAH grade. *n* = 15 per group. (c, d) Modified Garcia score and animal body weight evaluated at 1 d, 2 d, and 3 d after SAH modeling. ^∗^*p* < 0.05 vs. sham, ^#^*p* < 0.05 vs. SAH+vehicle, *n* = 15 per group. (e) Quantification of brain water content in the left hemisphere, right hemisphere, cerebellum, and brain stem at 24 h after SAH. ^∗^*p* < 0.05 vs. sham, ^#^*p* < 0.05 vs. SAH+vehicle, *n* = 6 per group. (f–h) Representative Western blot bands and densitometric quantification of tight junction proteins (ZO-1 and occludin). ^∗^*p* < 0.05 vs. sham. ^#^*p* < 0.05 vs. SAH+vehicle, *n* = 6 per group.

Subarachnoid blood clots were present along the circle of Willis at 24 h after SAH, and blood clots and hematoma showed obvious subsiding at 72 h after SAH **(**Figure 3(a)). There were no statistical differences in SAH grading scores between the CEP-treated group and the vehicle group at 1 day or 3 days after SAH (Figure 3(b)).

After CEP treatment, the aggravation of the neurological performance evaluated by the modified Garcia scale was significantly improved at 1 day and 2 days after SAH compared with the vehicle treatment; however, this improvement was not observed at 3 days after SAH (Figure 3(c)). The most obvious weight loss was observed at 2 days after SAH induction, and weight recovery in the CEP-treated group was significantly different compared with that in the vehicle group at 2 days and 3 days after SAH (Figure 3(d)). Interestingly, we observed a positive correlation between animal body weight and neurological function score (data not shown); therefore, we tried to use the mouse weight as an indicator to evaluate the severity of SAH injury.

Cerebral edema is an important factor leading to poor neurological function. In this study, the results of brain water content (BWC) analysis suggested that brain edema existed in the left and right hemispheres in the SAH+vehicle group at 24 h after SAH when compared with the sham group (Figure 3(e)). In the left hemisphere, there was a significant increase in BWC in the vehicle group, which was further significantly reversed by CEP treatment (Figure 3(e)). BBB dysfunction is the main cause of brain edema, and data from Western blotting showed that the expressions of zonula occludens-1 (ZO-1) and occludins were obviously decreased at 3 days after SAH. CEP treatment effectively reversed these harmful changes **(**Figures 3(f)–3(h)).

### 3.3. CEP Suppressed Lipid Peroxidation after SAH, but Not by Regulating the Expression of GPx4 and xCT

**Figure 4:**
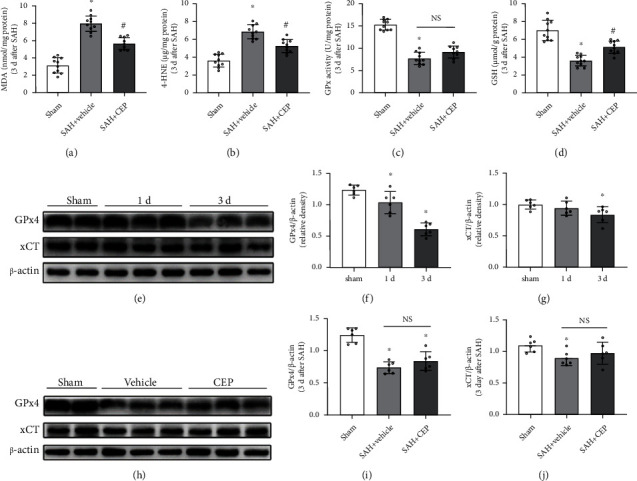
The effects of CEP on lipid peroxidation metabolites and GPx4/GSH antioxidant system after SAH. (a–d) MDA and 4-HNE contents, GPx4 activity, and GSH content measured by respective commercial kits at 3 d after SAH. ^∗^*p* < 0.05 vs. sham, ^#^*p* < 0.05 vs. SAH+vehicle, *n* = 8 per group. (e–g) Representative Western blot bands and densitometric quantification showed the time-dependent downregulation of GPx4 and xCT protein level after SAH. ^∗^*p* < 0.05 vs. sham, ^#^*p* < 0.05 vs. SAH+vehicle, *n* = 6 per group. (h–j) Representative Western blot bands and densitometric quantification showed rescue effect of CEP on GPx4 and xCT expression. ^∗^*p* < 0.05 vs. sham, ^#^*p* < 0.05 vs. SAH+vehicle, *n* = 6 per group.

Lipid peroxidation is an inadequate but necessary event during ferroptosis. Therefore, we evaluated the level of lipid peroxidation and antioxidant activity after SAH as an indicator of ferroptosis. MDA and 4-HNE, the end products of lipid peroxidation, were markedly accumulated in the SAH+vehicle group compared with those in the sham group at 3 days after SAH (Figures 4(a) and 4(b)), whereas GSH content and GPx4 activity were significantly declined (Figures 4(c) and 4(d)). CEP served an effective protective role by reducing the accumulation of MDA and 4-HNE and recovering the declined GSH content (Figures 4(a), 4(b), and 4(d)). GPx4 and xCT play an important role in resisting lipid peroxidation [29, 30]. However, CEP treatment was showed no effective recovery of GPx4 activity in SAH mice after three consecutive days of CEP treatment (Figure 4(c)). In correspondence, the expression levels of GPx4 and xCT decreased at 1 day and 3 days after SAH compared with the levels in the sham group, and the difference was significant at 3 days after SAH (Figures 4(e)–4(g)). CEP treatment improved the expression levels of GPx4 and xCT when compared with vehicle alone, but the difference was not statistically significant (Figures 4(h)–4(j)).

### 3.4. CEP Treatment Decreased the Expression Levels of ALOX15 in Endothelial Cells and Microglia *In Vivo*

**Figure 5:**
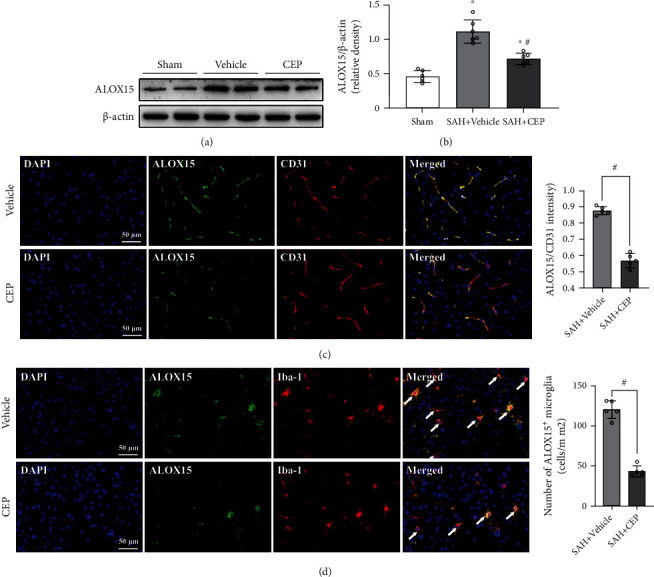
The effects CEP on ALOX15 expression inhibition after SAH. (a, b) Representative Western blot bands and densitometric quantification of ALOX15. ^∗^*p* < 0.05 vs. sham, ^#^*p* < 0.05 vs. SAH+vehicle, *n* = 6 per group. (c) Images of ALOX15/CD31 staining in the left hemisphere and quantitative analysis. ^#^*p* < 0.05 vs. SAH+vehicle, scale bar = 50 *μ*m, *n* = 5 per group. (d) Images of ALOX15 and Iba-1 immunostaining and quantitative analysis of ALOX15-positive microglia. ^#^*p* < 0.05 vs. SAH+vehicle, scale bar = 50 *μ*m, *n* = 5 per group.

ALOX15 is one of the key enzymes forming lipid peroxidation products. Data from Western blotting indicate that the increased levels of ALOX15 after SAH can be significantly suppressed by treatment with CEP (Figures 5(a) and 5(b)). Consistently, immunofluorescence staining results showed that the colocalization of ALOX15 with endothelial cells and microglia reduced significantly after CEP treatment compared with treatment with the vehicle alone (Figures 5(c) and 5(d)). The abovementioned evidence suggested that CEP treatment effect on lipid peroxidation inhibition may have been achieved by the suppression of the ALOX15 expression.

### 3.5. CEP Alleviated Characteristically Ferroptotic Mitochondrial Damage in Microglia and Endothelial Cells after SAH

**Figure 6:**
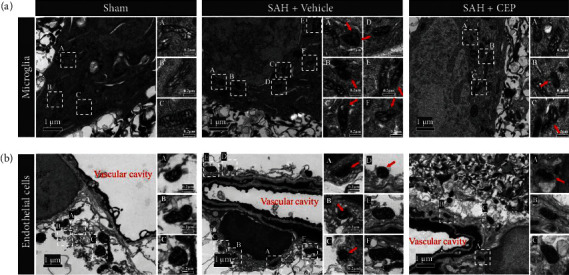
The mitochondrial damage in microglia and endothelial cells after SAH and the rescue effects of CEP. (a, b) Representative TEM picture of mitochondria in microglia and endothelial cells. Microglia presented irregular nuclear morphology (bean or jagged-shaped), electron-dense cytoplasm (dark cytoplasm), and distinct heterochromatin pattern (thick and dark chromatin condensation beneath the nuclear membrane). Endothelial cells were determined by typical vascular structure and its internal residual blood components. The red arrow points to the shrunken mitochondria with increased mitochondrial membrane density, vanishing cristae, and collapse of outer membrane. The white dashed frame marked with capital letters in the left overall image (scale bar = 1 *μ*m) corresponds to the scattered images (scale bar = 0.2 *μ*m) enlarged on the right.

**Figure 7:**
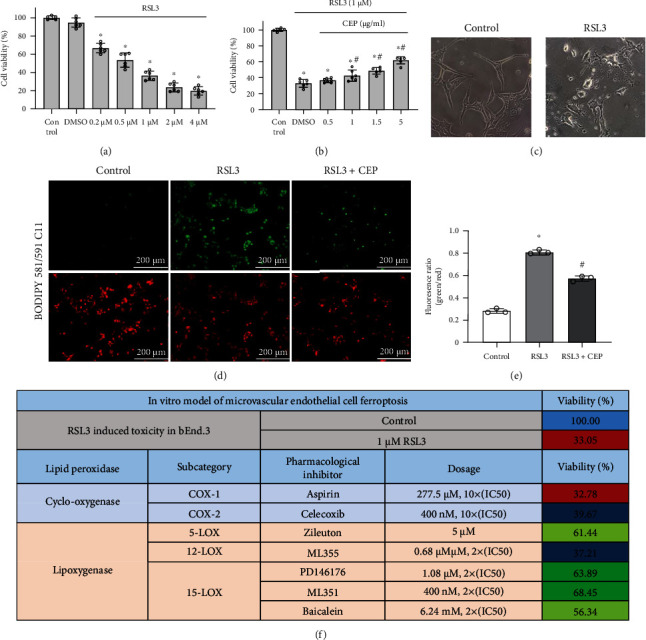
CEP increased cell viability and inhibited lipid peroxidation of bEnd.3 at 24 h after RSL3 exposure, and systematic inhibition of AA metabolizing enzymes identified ALOX15 as a target for ferroptosis. (a) RSL3 decreased cell viability detected by CCK-8. ^∗^*p* < 0.05 vs. control, *n* = 6 per group. (b) The CCK-8 results depicted that CEP saves RSL3-induced cell death in a concentration-dependent manner. ^∗^*p* < 0.05 vs. control, ^#^*p* < 0.05 vs. DMSO group, *n* = 6 per group. (c) Representative morphological images of bEnd.3 cells exposed to RSL3 with or without CEP. Scale bar = 20 *μ*m. (d, e) Representative picture of BODIPY 581/591 C11 staining and quantitative fluorescence ratio analysis of green (representing lipid peroxidation)/red (conventional coloring). (f) The table clarified that inhibitors of 15-lipoxygenase and 5-lipoxygenase, but not other types of oxygenase which catalyze AA, suppressed RSL3-induced ferroptosis in bEnd.3 cells.

Currently, morphological changes in mitochondria are the most convincing evidence of ferroptosis. Therefore, TEM was introduced to confirm that microglia and endothelial cells undergo ferroptosis after SAH. First, microglia were distinguished from other cell types by their small size, irregular nuclear morphology (bean or jagged-shaped), characteristically electron-dense cytoplasm (dark cytoplasm showed in the image), and distinct heterochromatin pattern (thick and dark chromatin condensation beneath the nuclear membrane) **(**Figure 6(a)). Endothelial cells as the innermost structure of blood vessels were determined by typical vascular structure and its internal residual blood components **(**Figure 6(b)). Representative images showed morphological changes indicating remarkable injury in the microglial mitochondria in the vehicle group with characteristics such as classical shrunken mitochondria with increased mitochondrial membrane density, vanishing cristae, and collapsing of the outer membrane **(**Figures 7(a) and 7(b)). This result suggests that ferroptosis at least partly occurred in the microglia after SAH. More importantly, this mitochondrial damage was reduced and the proportion of normal mitochondria was increased after CEP treatment compared with vehicle treatment (Figures 7(a) and 7(b)).

### 3.6. CEP Prevented RSL3-Induced Endothelial Cell Ferroptosis via Neutralizing ALOX15-Mediated Toxic Lipid Accumulation *In Vitro*

**Figure 8:**
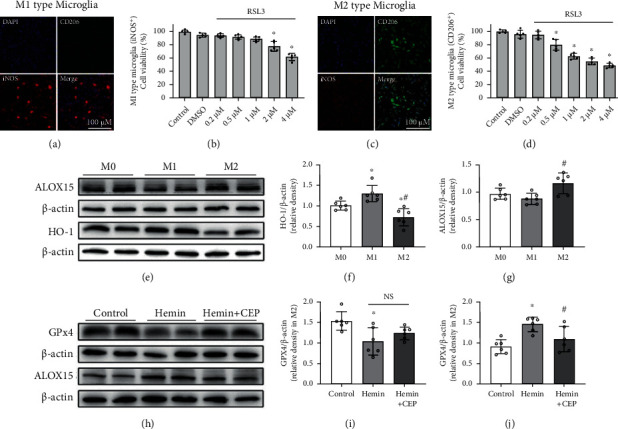
The sensitivity of different types of microglia to ferroptosis, and the effect of CEP on the expression of ALOX15 and GPx4 in hemin-induced BV2*in vitro*. (a, c) Representative images of iNOS and CD206 immunostaining in different polarization types of BV2 cells. (b, d) RSL3 decreased cell viability in M1- (b) and M2- (d) type microglia detected by CCK-8. ^∗^*p* < 0.05 vs. control, *n* = 6 per group. (e–g) Representative Western blot bands and densitometric quantification showed the protein level of ALOX15 and HO-1 in different polarization types of microglia. ^∗^*p* < 0.05 vs. M0 group, ^#^*p* < 0.05 vs. M1 group, *n* = 6 per group. (h–j) Representative Western blot bands and densitometric quantification showed the effect of CEP on GPx4 and ALOX15 expression in hemin-treated M2-type microglia. ^∗^*p* < 0.05 vs. control, ^#^*p* < 0.05 vs. hemin, *n* = 6 per group.

Based on the above demonstrated result that GPx4 was significantly deprived after SAH *in vivo*, we tried to simulate endothelial cell ferroptosis *in vitro* with a direct inhibitor of GPx4, RSL3, which is a widely used specific ferroptosis inducer. CCK-8 was introduced to evaluate cell viability after different treatments. RSL3 treatment caused a significant dose-dependent decline in viability in the bEnd.3 cell line compared with DMSO treatment (Figure 8(a)) (morphological changes are shown in Figure 8(c)). With different concentrations of CEP (0.5, 1, 2.5, and 5 *μ*g/mL) treatment, the viability of cells increased gradually, and the difference was significant compared with the DMSO group when the dose exceeded 1 *μ*g/mL (Figure 8(b)). To determine the extent of lipid peroxidation in endothelial cells, BODIPY 581/591 C11, an efficient lipophilic ROS sensor, was introduced to detect lipid ROS. Under the induction of RSL3, a large amount of lipid ROS accumulated (green fluorescence) on cells. Additional CEP treatment effectively reduced green/red fluorescence ratio compared with the RSL3 group, indicating a reduction in lipid peroxidation (Figures 7(d) and 7(e)).

PUFAs can be catalyzed by various oxygenases into peroxidized lipids. To determine the key role of ALOX15 lipoxygenases in ferroptotic signal formation, we introduced chemical inhibitors against diverse oxygenases and conducted a systematic cell viability experiment. The results showed that the specific inhibitors of two cyclooxygenases COX-1 (aspirin) and COX-2 (celecoxib) failed to rescue RSL3-induced ferroptosis even with concentrations up to 10 times those of the IC50. However, apart from 12-lox (ML355), selective inhibitors of other lipoxygenases such as 5-lox (zileuton), and 15-lox (PD146176, ML351, and baicalein) significantly protected cells from RSL3 toxicity at concentrations of two times the IC50 (Figure 7(f)). This result showed that 5/15-lox-mediated lipid peroxidation may be necessary for RSL3-induced endothelial cell ferroptosis. Thus, compatible with the *in vivo* results, cellular immunofluorescence showed the rescue effect of CEP and ML351 on ferroptosis *in vitro*.

### 3.7. M2-Type Microglia Were More Sensitive to Ferroptosis than the M1 Type, and CEP Reversed ALOX15 and GPx4 Changes in Hemin-Treated M2 Microglia

As ferroptosis occurring in different polarization types of microglia may probably lead to completely different neurological outcomes after SAH, we induced M1- and M2-type microglia to study their sensitivity to ferroptosis and potential mechanisms. Cellular immunofluorescence separately showed iNOS-positive M1-type microglia (Figure 8(a)) and CD206-positive M2-type microglia (Figure 8(c)). Results of the CCK-8 assay suggested that M2-type microglia suffered more severe cytotoxicity than the M1 type with the same concentration of RSL3 (Figures 8(b) and 8(d)). To explain this phenomenon, we tested a variety of factors related to peroxidation and antioxidation systems. Western blotting results indicated that the M2-type microglia expressed more ALOX15 and less heme oxygenase-1 (HO-1) compared with the M1 type (Figure 8(e)–8(g)). On hemin inducement, the expression of GPx4 in M2-type microglia was significantly decreased, whereas that of ALOX15 was markedly increased. CEP treatment reversed both these changes (Figures 8(h)–8(j)), but only inhibition on ALOX15 expression showed a statistically significant difference (Figures 8(h) and 8(j)).

## 4. Discussion

Ferroptosis is a recently determined form of a type of programmed cell death that plays a vital role in tumor suppression and embryonic development [8, 31]. With the gradual elucidation of its mechanism, ferroptosis has been wildly recognized as the type of pathological cell death implicated in acute diseases (i.e., stroke, traumatic brain injury, and acute lung injury) as well as in degenerative diseases (i.e., Parkinson's disease, Alzheimer's disease, and macular degeneration) [15, 20, 32–35]. Hemorrhagic strokes seem to be closely related to ferroptosis as they are vulnerable to iron-enriched hemoglobin metabolites released from lysed erythrocytes. This association has been demonstrated in several studies [12–14]; however, almost all of these studies assume that the cell type in which ferroptosis occurs is neurons but not others. In this present study, we provided evidence that endothelial cells and microglia undergo ferroptosis, which may be facilitated by upregulated ALOX15 after SAH, in an SAH model both *in vitro* and *in vivo*. These results were supported by several published studies, which demonstrated that upregulated ALOX15 induced ferroptosis in asthmatic epithelial cells *in vivo* [36] and macrophages (RAW 264.7 and bone marrow-derived macrophages) and microglia (EOC 20) *in vitro* [37]. Additionally, CEP, a type of BBIQ alkaloid, protected the brain from SAH-related injury, and this effect was achieved at least in part by inhibiting the ALOX15-mediated endothelial cell and microglial ferroptosis.

Iron deposition, lipid peroxidation accumulation, and lipophilic antioxidant system deficiency are critical factors associated with the induction of ferroptosis [31]. It is convincing, without fully being proven till now, that the generation of peroxygenated lipids combined with the GPx4/GSH antioxidant system defect is an indicator of ferroptosis [38]. The lipids that can be oxidized to form ferroptotic signals are PUFAs, including AA and AdA, with multiple oxidation sites. Recent studies have demonstrated that singly oxidized PUFA-OOH fails to mediate ferroptosis both *in vivo* and *in vitro*. Only when PUFAs were esterified into phospholipids (PLs) to form PLs-PUFA could they be subsequently oxidized to generate a ferroptotic death signal [18, 19]. During this process, acyl-CoA long-chain family member 4 (ACSL4) plays a critical acetylation role in PUFA-CoA formation, which was recently demonstrated to be implicated in ferroptosis during ICH and SAH [15, 39]. Several iron-containing enzymes and nonenzymatic processes (i.e., Fenton reaction) were reported to catalyze PUFA-CoA into PUFA-CoA-OOH [10, 31]. The lipoxygenase family especially ALOX15 has been extensively studied in recent years, and its connection with ferroptotic lipid signaling has been gradually clarified [18, 19, 40]. Kagan et al. have demonstrated that doubly and triply oxygenated PE-AA-15-OOH and PE-AdA-15-OOH, oxidized by ALOX15, were able to promote ferroptosis [18]. The data published by Wenzel et al. in *Cell* also authenticated same conclusions and named PE-AA-15-OOH as 15-HpETE-PE. In addition, they demonstrated that the catalytic effect of ALOX15 was strengthened as it was immobilized on the cell membrane by phosphatidylethanolamine-binding protein 1 (PEBP1). More importantly, they reported that 15-HpETE-PE was catalyzed by ALOX15 as a ferroptotic signal in their TBI model [19]. The pharmacological inhibitor of ALOX15, baicalein, also showed neuroprotective effect in ICH and TBI models [41, 42]. In this present study, we revealed that the protein level of ALOX15 started to increase at 24 h after SAH modeling and maintained a high level throughout the whole EBI period. Immunofluorescence staining results showed that elevated ALOX15 was mainly expressed in the microglia of the temporal cortex around the hemorrhage, whereas meaningful positive ALOX15 signals were hardly found in the brain slices in the sham group. Several previous studies have revealed the injury-promoting effect of ALOX15 in ischemic stroke and periventricular leukomalacia [43, 44]. The cell types where ALOX15 was highly expressed after injury varied in different CNS disease models, and neurons, microglia, and endothelial cells were the three main cell types reported [21, 43, 44]. Based on our data, ALOX15 was also significantly detected in microvascular endothelial cells over the whole injured hemisphere especially in the dorsal cortex opposite the bleeding point after SAH. More interestingly, we observed obvious scattered bleeding points in the dorsal cortex where ALOX15 was highly expressed in endothelial cells. Importantly, although we still cannot explain why SAH at the base of the skull can cause hemorrhage in the dorsal cortex, this result at least suggested that the high expression of ALOX15 in endothelial cells may lead to vascular damage or even hemorrhage. This speculation was supported by several previous studies, one of which demonstrated an increased expression of ALOX15 after middle cerebral artery occlusion- (MCAO-) mediated warfarin-associated hemorrhagic transformation (HT), and ALOX15 knockout or drug suppression significantly reversed this damage [21]. Another previous study further affirmed the involvement of endothelial cell ferroptosis in the hemorrhagic transformation of the type 2 male diabetic rat MCAO model. They revealed the occurrence of ferroptosis in endothelial cells by determining the strong vascular localization of ferroptosis marker iron-responsive element binding protein 2 (IREB2) and citrate synthase and by *in vitro* verification in primary brain microvascular endothelial cells [45]. Based on the abovementioned information, we speculated that ferroptosis may occur in microglial and endothelial cells with a high expression of ALOX15 and cause corresponding damage after SAH. Moreover, ALOX15 may become a potential marker for the identification of ferroptosis.

The identification of ferroptosis is a priority of this subject. So far, no specific marker molecules or staining methods that can accurately identify the occurrence of ferroptosis in tissues have been identified. The morphological changes in the mitochondria observed by TEM are the “gold standard” for identifying ferroptosis both *in vivo* and *in vitro* [8, 31]. We failed to systematically observe the mitochondria in microvascular endothelial cells because the probability of the presence of mitochondria in blood vessels in the sections we observed was pretty low. Fortunately, we successfully identified microglia based on its typical characteristics via TEM as described above [46]. It should be noted that it is unreasonable to use “shrunken mitochondria” alone as an indication of cell ferroptosis, as different cross-sections of mitochondria themselves show various sizes. Our data showed not only mitochondrial shrinkage but also obvious increase in mitochondrial membrane density, outer membrane rupture, and vanishing cristae at 3 days after SAH. Further, lipid peroxidation accumulation and defect in GPx4/GSH antioxidant system are indirect but important indicators of ferroptosis [38]. Our previous study pointed out that 3 days after SAH may be the peak point of lipid peroxidation accumulation [13]; accordingly, it was chosen as the main observation point. We used specific kits (MDA: #A003; 4-HNE: #H268; Jiancheng Bioengineering Institute, Nanjing, China) to measure the contents of the end products of lipid ROS: MDA and 4-HNE. The results suggested significant accumulation in the temporal cortex. In addition, GSH is a vital intracellular antioxidant synthesized from glutamate, cysteine, and glycine, wherein glutamate (from inside to outside of cell) and cysteine (from outside to inside of cell) were directionally transferred by SLC7A11 (xCT) in a 1 : 1 ratio. As cysteine availability limits the biosynthesis of GSH, xCT deficiency will lead to glutamate accumulation and GSH depletion. GPx4 can utilize GSH to eliminate the lipid peroxide form in PE-PUFAs [31, 38]. Although not rigorous enough, many animal model studies, including those on osteoporosis, TBI, and spinal cord injury, regard the lack of GPx4 as the landmark of ferroptosis [20, 29, 47]. In this present study, we revealed that the activity of GPx4 and the content of GSH in the temporal cortex were significantly decreased at 3 days after SAH. Consistently, Western blotting results showed obvious decline in the protein levels of GPx4 and xCT. Actually, multiple studies including our previous studies have demonstrated the occurrence of neuronal ferroptosis after SAH both in mouse and rat models. To the best of our knowledge, this present study, for the first time, demonstrates that ferroptosis occurs in the microglia after SAH.

As a type of BBIQ purified from Chinese herbal medicine *Stephania cepharantha*, CEP possesses a variety of pharmacological properties including antioxidative, anti-inflammatory, anticancer, antiviral, and antiparasitic properties [25]. CEP has been demonstrated to suppress the expression of proinflammatory cytokines in various *in vivo* and *in vitro* models [48]. The protective effects of CEP in the CNS are gradually being valued given its ability to cross the BBB. It was reported to suppress LPS-induced microglial activation characterized by the synthesis of iNOS and release of proinflammatory cytokines [49]. Further, CEP-hydrochloride was shown to have neuroprotective effects via the degeneration of specific toxicity receptors in an autophagic manner in an *in vitro* model of spinal and bulbar muscular atrophy [50]. Additionally, CEP was revealed to regulate the metabolism of AA by reducing the expression of COX-2 and ALOX15 in liver and brain ischemia/reperfusion models, respectively [26, 51]. Moreover, various specific properties suggest the potential antiferroptosis effects of CEP; these include direct radical scavenging-dependent antilipid peroxidation activity, efficient chelating ability of Fe^2+^, and amphipathic nature-facilitated cell membrane permeability and interaction with lipophilic compounds [25, 52]. Because lipid peroxides provide direct ferroptotic signals, Fe^2+^ is the predisposing factor of ferroptosis, and the ALOX15/PEBP1 complex catalyzes the formation of ferroptotic signals on PE-rich cell membranes. More importantly, another BBIQ, dauricine, which possesses a highly similar molecular structure and pharmacological properties to CEP, was recently reported to suppress ferroptosis after ICH [28]. Therefore, we introduced CEP in our study to explore its possible antiferroptotic and neuroprotective effects after SAH. Surprisingly, CEP was shown to rescue the neurological deficit and mice with weight loss. Brain edema and injured tight junction represented by reduced protein levels of ZO-1 and occludin were also reversed by CEP treatment. Although CEP failed to retrieve the decreased levels of GPx4 and xCT, it was surprisingly found to relieve ferroptotic mitochondrial changes in the microglia, eliminate the accumulation of the end products of lipid ROS, and reduce the expression of ALOX15 in the microglia and endothelial cells. For further verification the therapeutic effect of CEP, we preinjected recombinant ALOX15 into the ventricle and then intraperitoneally inject CEP to verify that CEP functions in a manner of depriving ALOX15 (Figure 1). Western bolt was used to detect the levels of ALOX15 and GPx4. Animal weight, brain water content, and Garcia score were detected to evaluate the neurological function of mice. In mice with high levels of ALOX15, the content of GPX4 was further reduced, and the neurological function of the mice was further deteriorated. CEP treatment can not only significantly reduce the content of ALOX15 but also rescue the reduction of GPX4 (Figure S1A-C). In addition to BWC, both animal weight and Garcia score show the therapeutic effect of CEP on neurofunction damage caused by high levels of ALOX15 (Figure S1D-F). These results suggest that CEP inhibits ferroptosis by suppressing ALOX15 expression and, finally, exerts neuroprotective effects.

For further identifying the occurrence of ferroptosis and understanding the mechanism of CEP in endothelial cells, we introduced the bEnd.3 cell line. As the protein level and activity of GPx4 were obviously declined in the SAH mouse model, RSL3, a direct GPx4 inhibitor, was adopted to simulate the GPx4-deficient environment *in vitro*. Results of CCK-8 demonstrated a RSL3 dose-dependent cell death trend, which was reversed by CEP treatment. Lipophilic ROS accumulation detected by a specific lipophilic sensor, BODIPY 581/591 C11, suggested that ferroptosis mainly occurred in a GPx4-deficient condition. Using a series of specific inhibitors, we demonstrated that pharmacological inhibition of ALOX5 or ALOX15 significantly reduced RSL3-induced cell death, whereas the inhibition of COX-1/2 and ALOX12 showed no obvious preservation. With the absence of mitochondrial changes observed by TEM as direct evidence, these results supplement the possibility of ferroptosis occurring in endothelial cells in mice with SAH with GPx4 deficiency.

Microglia, as the resident macrophages in CNS, can be activated to M1 and M2 two phenotypes after various diseases including SAH [53]. In general, the M1 type is considered to be neurotoxic because of its inflammation-promoting effect, whereas the M2 type is generally supposed to be neuroprotective. Therefore, ferroptosis occurring in different types of microglia may lead to completely different neurological outcomes after SAH. We introduced BV2 here to further investigate the sensitivity of different types of microglia to ferroptosis and the underlying potential mechanism. Our data showed that M2 microglia was more vulnerable to RSL3-induced ferroptosis. After detecting a variety of factors related to peroxidation and antioxidation, we found that ferroptosis may be associated with increased prooxidant ALOX15 and decreased antioxidant HO-1 in M2 microglia when compared with those in the M1 type. Of course, more research needs to be conducted to verify this hypothesis. Furthermore, M2-type BV2 were treated with hemin (Fe3+ protoporphyrin IX), the lysate of erythrocytes to simulate SAH *in vitro*. After treatment with hemin, the ALOX15 expression was significantly increased and GPx4 was markedly decreased in M2 microglia. These results implicated that M2 microglia are highly likely to undergo ferroptosis with the stimulation of erythrocyte lysate *in vivo*, because RSL3 is the direct inhibitor of GPx4 and hemin induces a significant reduction in GPx4. Moreover, ferroptosis may also be facilitated by increased ALOX15 expression.

However, there are still some limitations to this work that cannot be ignored. There is evidence similar to the colocalization of ALOX15 and apoptosis-inducing factor (AIF) proving the correlation between ALOX15 and apoptosis [43]. We cannot rule out the involvement of apoptosis in the neuroprotective effect of ALO15 downregulation by CEP treatment. This is also a common dilemma in the research on ferroptosis-related disease models, because of the diversity of cell death forms. Moreover, the investigation of the mechanism that specifically affects neurological function after microglial ferroptosis is lacking in this study, although many studies, including our previous studies, have shown that inhibiting ferroptosis with specific inhibitors can suppress the release of proinflammatory factors after injury. Finally, animal experiments were only conducted in adult male mice, and we still cannot rule out the influence of gender and age on the results of the experiment.

## 5. Conclusion

In summary, our study, mainly as a proof-of-concept research, for the first time identified the occurrence of ferroptosis in the microglia during EBI in SAH and in the endothelial cells using an *in vitro* GPx4 deficiency model. Furthermore, CEP treatment significantly reversed cell death following ferroptosis induction *in vitro* and rescued the neurological injury after SAH, at least partially, through inhibiting microglia and endothelial cell ferroptosis.

## Data Availability

The data set generated in this study is available from the corresponding author upon request.

## Abbreviations

SAH: Subarachnoid hemorrhage
ALOX15: 15-Lipoxygenase-1
CEP: Cepharanthine
GPx4: Glutathione peroxidase 4
GSH: Glutathione
EBI: Early brain injury
BBB: Blood–brain barrier
CNS: Central nervous system
PUFA: Polyunsaturated fatty acids
AA: Arachidonic acid
12/15(S)-HETE: 12/15(S)-Hydroxyicosatetraenoic
PE: Phosphatidylethanolamines
PFA: Paraformaldehyde
PBS: Phosphate-buffered saline
TEM: Transmission electron microscopy
MDA: Malondialdehyde
4-HNE: 4-Hydroxynonenal
ZO-1: Zonula occludens-1
HO-1: Heme oxygenase-1.

## Supplementary Materials

Supplementary Materials Supplementary Figure S1: effect of CEP on ALOX15 high level mice. (A–C) Representative western blot bands and densitometric quantification of ALOX15 and GPx4. ^∗^*p* < 0.05 vs. sham, ^#^*p* < 0.05 vs. SAH+vehicle, ^$^*p* < 0.05 vs. SAH+rALOX15. *n* = 6 per group. (D–F) Animal body weight, modified Garcia score, and brain water content, respectively, evaluated at 2 d, 3 d, and 1 d after SAH modeling. ^∗^*p* < 0.05 vs. sham, ^#^*p* < 0.05 vs. SAH+vehicle, ^$^*p* < 0.05 vs. SAH+rALOX15. *n* = 6 per group.
