# Correction to “Cepharanthine Attenuates Early Brain Injury after Subarachnoid Hemorrhage in Mice via Inhibiting 15‐Lipoxygenase‐1‐Mediated Microglia and Endothelial Cell Ferroptosis”

**DOI:** 10.1155/omcl/9890415

**Published:** 2025-12-15

S. Gao, L. Zhou, J. Lu, Y. Fang, H. Wu, W. Xu, Y. Pan, J. Wang, X. Wang, J. Zhang, and A. Shao, “Cepharanthine Attenuates Early Brain Injury after Subarachnoid Hemorrhage in Mice via Inhibiting 15‐Lipoxygenase‐1‐Mediated Microglia and Endothelial Cell Ferroptosis,” *Oxidative Medicine and Cellular Longevity* 2022, no. 1 (2022): 4295208, https://doi.org/10.1155/2022/4295208.

In the article titled “Cepharanthine Attenuates Early Brain Injury after Subarachnoid Hemorrhage in Mice via Inhibiting 15‐Lipoxygenase‐1‐Mediated Microglia and Endothelial Cell Ferroptosis,” there was an error in Figure 5d related to an accidental duplication of the panels representing vehicle‐treated microglia. The overlapping images were included in error during figure preparation and Figure 5d is corrected as follows:

**Figure 5:**
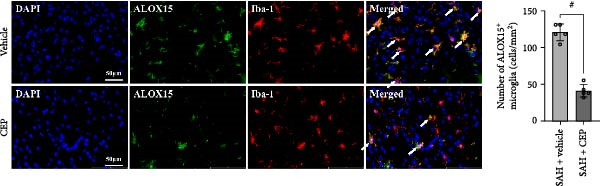
(d) Images of ALOX15 and Iba‐1immunostaining and quantitative analysis of ALOX15‐positive microglia. #*p* < 0.05 vs. SAH + vehicle, scale bar = 50 μm, *n* = 5 per group.

We apologize for this error.

