# Supplementary figures and images for: Cepharanthine Attenuates Early Brain Injury after Subarachnoid Hemorrhage in Mice via Inhibiting 15-Lipoxygenase-1-Mediated Microglia and Endothelial Cell Ferroptosis
Source: Oxid Med Cell Longev. 2022 Feb 9;2022:4295208. doi: 10.1155/2022/4295208 (PMC8850040; doi:10.1155/2022/4295208)

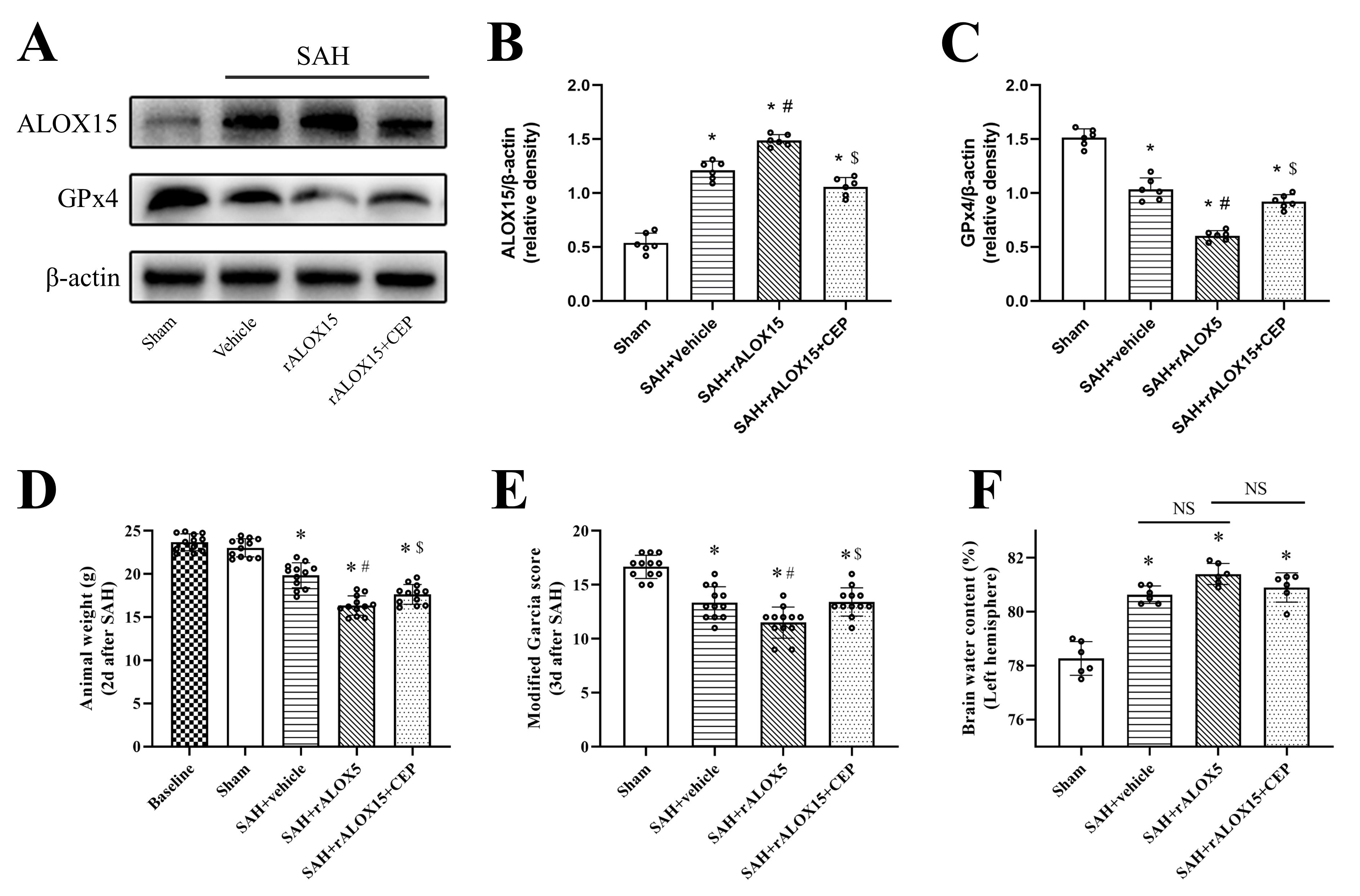


**Supplementary Figure 1**

Supplement: Supplementary Materials — Supplementary Figure S1: effect of CEP on ALOX15 high level mice. (A–C) Representative western blot bands and densitometric quantification of ALOX15 and GPx4. ∗p < 0.05 vs. sham, #p < 0.05 vs. SAH+vehicle, $p < 0.05 vs. SAH+rALOX15. n = 6 per group. (D–F) Animal body weight, modified Garcia score, and brain water content, respectively, evaluated at 2 d, 3 d, and 1 d after SAH modeling. ∗p < 0.05 vs. sham, #p < 0.05 vs. SAH+vehicle, $p < 0.05 vs. SAH+rALOX15. n = 6 per group. [file 4295208.f1.docx]
